# Hydrogels Synthesized by Electron Beam Irradiation for Heavy Metal Adsorption

**DOI:** 10.3390/ma10050540

**Published:** 2017-05-18

**Authors:** Elena Manaila, Gabriela Craciun, Daniel Ighigeanu, Catalina Cimpeanu, Catalina Barna, Viorel Fugaru

**Affiliations:** 1 National Institute for Laser, Plasma and Radiation Physics, Electron Accelerators Laboratory, #409 Atomistilor St., Magurele 077125, Romania; elena.manaila@inflpr.ro (E.M.); daniel.ighigeanu@inflpr.ro (D.I.); 2 Horia Hulubei National Institute for Physics and Nuclear Engineering, 30 Reactorului St., Magurele 077125, Romania; catalinac@nipne.ro (C.C.); catalina.barna@nipne.ro (C.B.); vfugaru@nipne.ro (V.F.)

**Keywords:** hydrogels, electron beam irradiation, cross-linking, trimethylolpropane trimethacrylate

## Abstract

Poly(acrylamide co-acrylic acid) hydrogels were prepared by free-radical copolymerization of acrylamide and acrylic acid in aqueous solutions using electron beam irradiation in the dose range of 2.5 kGy to 6 kGy in atmospheric conditions and at room temperature. The influence of the absorbed dose, the amount of cross-linker (trimethylolpropane trimethacrylate) and initiator (potassium persulfate) on the swelling properties and the diffusion coefficient and network parameters of hydrogels were investigated. The structure and morphology of hydrogels were characterized by Fourier Transform Infrared Spectroscopy (FTIR) and Scanning Electron Microscopy (SEM). The use of the obtained hydrogels by the removal of Cu^2+^ and Cr^6+^ from aqueous solutions was investigated at room temperature. During the adsorption of metal ions on hydrogels, the residual metal ion concentration in the solution was measured by an atomic absorption spectrophotometer (AAS). It has been established that the use of a relatively small amount of trimethylolpropane trimethacrylate for hydrogel preparation has led to the increasing of swelling up to 8500%.

## 1. Introduction

Heavy metal ions are harmful and toxic to human beings and the environment. Therefore, they must be removed from wastewater and drinking water. The main threats to human health from heavy metals (HM) are associated with exposure to cadmium (Cd), lead (Pb), mercury (Hg) and arsenic (As) (arsenic is a metalloid, but is usually classified as a HM), but additionally, there are other elements known as HM: antimony (Sb), bismuth (Bi), cerium (Ce), chromium (Cr), cobalt (Co), copper (Cu), gallium (Ga), gold (Au), iron (Fe), manganese (Mn), molybdenum (Mo), nickel (Ni), platinum (Pt), silver (Ag), tellurium (Te), thallium (Tl), tin (Sn), uranium (U), vanadium (V) and zinc (Zn) [1]. Some heavy metals (e.g., Fe, Zn, Cu, Co, Mn and Mo) in small quantities are nutritionally essential for the maintenance of human metabolism [2]. However, heavy metals are also very toxic and non-biodegradable; hence, they can readily be accumulated in an ecological system, induce irreversible pollution, or cause adverse health effects in the human body [3]. Because they are non-biodegradable and can accumulate in the food chain, heavy metal ions from industrial effluents seriously threaten the environment and public health, even at very low concentrations [4,5,6]. Increasing effort has been concentrated on developing various methods that can effectively remove heavy metal ions from aqueous environments [7]. Some widespread methods for removing heavy metals include chemical coagulation and precipitation [8,9], membrane filtration [10,11], ion exchange [12,13] and electrochemical technologies [14]. They have been developed for the early detection and removal of toxic heavy metals from environments, but limited success has been achieved to date due to their low efficiency and the further generation of toxic sludge or other waste products. Additionally, these methods require expensive equipment and involve time-consuming and laborious procedures [15]. Alternatively, adsorption offers many potential advantages for the removal of toxic heavy metals over other methods, thanks to its flexibility in design and operation, high-quality treated effluent, reversible nature for multiple uses and many commercially available adsorbent materials, such as activated carbon [16,17], zeolite [18], clay [19,20], lignin, chitosan and other polymer adsorbents [7,21]. Compared to the conventional adsorbent materials above, hydrogel-based adsorbents have attracted special attention due to their high potential for the effective removal of heavy metals [15,22]. Hydrogels usually have physically well-defined three-dimensional porous structures and chemically responsive functional groups, which enable one to readily capture metal ions from wastewater and to release and clear them upon changes in aqueous solution conditions [15,23]. The hydrophilic character of hydrogel adsorbents enables them to form a flexible network of polymer chains, which allows metal ions to quickly penetrate into the water network and form stable complexes with functional groups [15,24]. Various techniques are used for hydrogel preparation [25], such as physical and chemical cross-linking [26,27], grafting polymerization [28] and radiation cross-linking [29,30]. The radiation technique is more preferable than the chemical one, because of the advantage offered by the gentle control of cross-linking level by variation of the absorbed dose. In some studies, hydrogels have been obtained by simultaneous free radical copolymerization and cross-linking in the presence of polyfunctional monomers with or without the use of radiation techniques [31,32,33,34].

This study carries on from previously performed research [35]. The poly(acrylamide co-acrylic acid) hydrogels were prepared by free-radical copolymerization of acrylamide and acrylic acid in aqueous solutions using electron beam irradiation in the dose range of 2.5 kGy to 6 kGy in atmospheric conditions and at room temperature. The influence of the absorbed dose, the amount of cross-linker (trimethylolpropane trimethacrylate) and the initiator (potassium persulfate) on the swelling properties, the diffusion coefficient and the network parameters of hydrogels was investigated. The structure and morphology of hydrogels were characterized by Fourier Transform Infrared Spectroscopy (FTIR) and Scanning Electron Microscopy (SEM). The use of the obtained hydrogels by the removal of Cu^2+^ and Cr^6+^ from aqueous solutions was investigated at room temperature. During the adsorption of metal ions on hydrogels, the residual metal ion concentration in the solution was measured by an atomic absorption spectrophotometer (AAS).

## 2. Experimental

### 2.1. Materials

The materials used for experiments are shown in Table 1. Acrylamide (AMD), acrylic acid (AA), potassium persulfate (PP, as initiator) and trimethylolpropane trimethacrylate (TMPT, as cross-linker), were purchased from Sigma Aldrich (Redox Group Company, Buchares, Romania) and were used without further purification.

The metal ions Cu^2+^ and Cr^6+^ were provided as CuSO_4_ and K_2_Cr_2_O_7_ by Sigma Aldrich (Redox Group Company, Bucharest, Romania). These reagents were of analytical grade and prepared with double-distilled water.

### 2.2. Experimental Installation and Sample Preparation

The hydrogels were obtained by irradiation using an electron beam accelerator called ALID-7, in atmospheric conditions and at a room temperature of 25 °C. ALID-7 was built in the Electron Accelerators Laboratory from the National Institute for Lasers, Plasma and Radiation Physics, Bucharest, Romania. ALID-7 is a linear electron accelerator of travelling-wave type, operating at a wavelength of 10 cm. The accelerating structure is a disk-loaded tube operating in the π/2 mode. The optimum values of the electron beam (EB) parameters, namely peak current I_EB_ and EB energy E_EB_, to produce maximum output power P_EB_ for a fixed pulse duration *τ*_EB_ and repetition frequency f_EB_ are as follows: E_EB_ = 5.5 MeV, I_EB_ = 130 mA, P_EB_ = 670 W (f_EB_ = 250 Hz, *τ*_EB_ = 3.75 μs).

The EB effects are related to the absorbed dose (D) and absorbed dose rate (D*). The absorbed dose is the major parameter in the electron beam irradiation [36]. The performances of polymerization and copolymerization processes are provided by the strict control of this parameter [36]. In our experiments the electron beam dose rate was fixed at 2 kGy/min in order to accumulate doses between 2.5 kGy and 6 kGy. The absorbed dose was determined using the graphite calorimeter and conventional Fricke dosimeter. An important step was the establishment of the electron beam penetration depth in the sample, in order to ensure equal doses at the entry and exit of the irradiated sample. The thickness requirements of the material were calculated from the following relationship [37]:

(1) E=2.6×t×ρ+0.3,

where *E* [MeV] is the beam energy, in our case 5.5 MeV; *t* [cm] is the thickness; and *ρ* [g·cm^–3^] is the sample density, in our case 1 g·cm^−3^.

The thickness of the irradiated samples was set at 20 mm. For irradiation, two types of aqueous solutions were prepared (Table 2). The solutions were placed in polyvinylchloride (PVC) containers with a 3 cm diameter and irradiated using electron beam irradiation in the dose range of 2.5 kGy to 6 kGy in atmospheric conditions and at a room temperature of 25 °C.

After irradiation, the obtained hydrogels were cut into pieces of 3–4 mm in length, dried in the air for three days and then in a laboratory oven at 50 °C for 12 h to reach a constant weight and then stored in desiccators. The dried hydrogels were used to determine the parameters of swelling, diffusion and network.

### 2.3. Measurements

Before making any measurements, the hydrogels were appraised visually as ductile materials. During the tests, all the samples used remained intact; they did not break or shatter.

Experiments of swelling and network studies: The swelling of dried hydrogels was carried out by immersion in double-distilled water at 25 ± 0.1 °C in a water bath. After wiping with filter paper at 24, 48, 72 and 96 h, the amount of absorbed water was determined by weighing the samples with an electronic balance (HR 200, 0.1 mg resolution). The experiments were carried out in triplicate and the data were reported as average values.

Metal ion uptake studies: Uptake experiments of metal ions at different pHs were achieved using the hydrogels as sorbents. Sorption was carried out by immersion of 0.5 g of each dried hydrogel in 100 mL aqueous media containing varying concentrations of ions of Cu^2+^ and Cr^6+^, for 72 h at room temperature (25 ± 0.1 °C). The pH was adjusted using 0.1 M NaOH and 0.1 M HCl. For samples that showed maximum uptake, the residual concentration of metal ions was measured using a GBC Avanta Σ atomic absorption spectrometer (AAS) (GBC Scientific Equipment Pty Ltd/Victoria, Australia). Chromium and copper determination methods had the following parameters: Cr—425.4 nm wavelength, 0.17 μg/mL sensitivity, 6 mA lamp current, air-acetylene flame and Cu—324.7 nm wavelength, 0.025 μg/mL sensitivity, 3 mA lamp current, air-acetylene flame.

FTIR analysis: In order to obtain spectral information regarding the chemical structure of hydrogels, a FTIR spectrophotometer—Bruker Tensor 27 (Bruker, Bremen, Germany) was used by the ATR measurement method. Samples’ spectra are the average of 30 scans realized in absorption, in the range of 4000–600 cm^−1^, with a resolution of 4 cm^−1^.

Scanning Electron Microscopy (SEM): The morphology of the lyophilized hydrogels was determined using a scanning electron microscope (FEI/Phillips, Hillsboro, OR, USA). Small pieces of swelled gels were freeze-dried in order to avoid the collapse of the porous structure. Freeze-drying was carried out in a vacuum at −80 °C for 40 h using the Christ Alfa 2–4 (Martin Christ, Osterode am Hartz, Germany) lyophilizer. For scanning, the lyophilized samples have been cut in order to expose the inner surface. Samples were placed on an aluminum mount, sputtered with gold palladium and then scanned at an accelerating voltage up to 30 kV.

## 3. Results and Discussion

### 3.1. Swelling and Diffusion Experiments

#### 3.1.1. The Measurement of Gel Content

The hydrogel content of a given material is estimated by measuring its insoluble part in dried samples after immersion in water. To perform measurements, dried samples of 0.05 g were immersed in double-distilled water in order to swell for 72 h [38]. After filtration, the extracted gel was dewatered with a non-solvent ethanol, dried out in the air and then in a laboratory oven for 5 h at 70 °C and finally reweighed. The gel content was calculated as follows:

(2) $$Gel(\%)=\frac{W_{1}}{W_{0}}\times 100$$

where *W*_0_ is the initial weight of the dried sample and *W*_1_ is the weight of sample after extraction from the water and drying.

**Figure 1:**
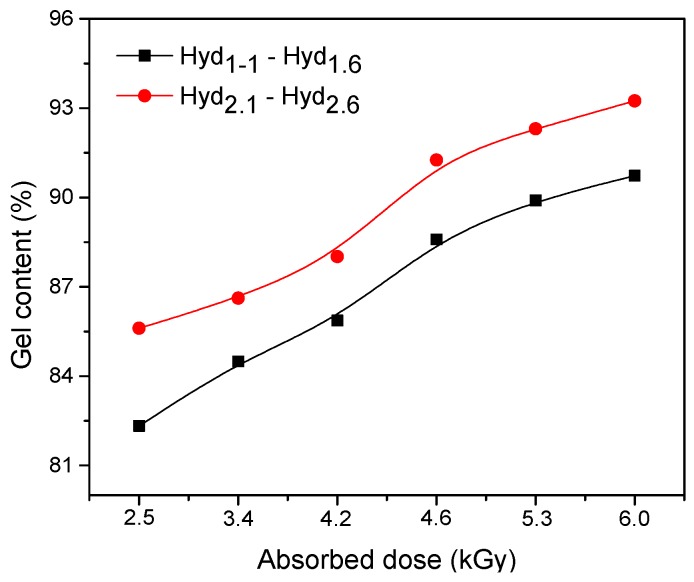
The effect of the absorbed dose on the gel fraction of hydrogels.

In Figure 1 is presented the effect of the electron beam absorbed dose, cross-linking agent (TMPT) and initiator (PP) amount on the hydrogels’ gel content. For a low dose of 2.5 kGy, the gel content is over 80% for both hydrogel types, Hyd_1_ and Hyd_2_, which differ by the amount of TMPT and PP. For both hydrogel types, the gel content increases with an increase in the absorbed dose. About 90% gel content is attained at doses higher than 5.3 kGy. Also, the gel content depends on the concentration of initiator and cross-linker. It has been observed that, when increasing the TMPT and PP amount, the gel content increases. If the concentration of TMPT (cross-linking agent) increases, there will be more cross-linking so that the gel content increases.

#### 3.1.2. Degree of Swelling

Furthermore, dynamic swelling experiments have been performed on the cross-linked AMD/AA/TMPT hydrogels in double-distilled water, at room temperature (25 ± 0.1 °C), in order to determine the maximum water uptake. The mass increase was pursued as a function of time. Also, all the samples were weighed at 24, 48 and 72 h. This determination was absolutely necessary in order to establish the time of immersion of hydrogels in aqueous solutions with heavy metals.

The water uptake, expressed in percentage, was calculated via the following equation [34,39]:

(3) $$S(\%)=\frac{M_{t}-M_{0}}{M_{0}}\times 100$$

where *M_t_* is the mass of the swollen gel at time *t* and *M*_0_ is the initial mass of the dried gel (at time *t* = 0).

**Figure 2:**
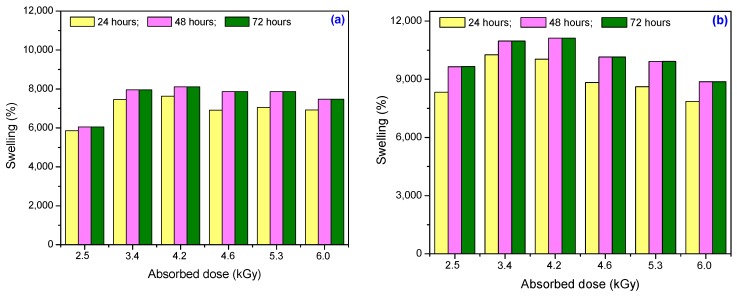
The effect of the absorbed dose on the hydrogels’ water uptake at 24, 48 and 72 h: (**a**) 3.70 × 10^−3^ mol/L PP and 2.95 × 10^−3^ mol/L TMPT; (**b**) 7.40 × 10^−3^ mol/L PP and 5.90 × 10^−3^ mol/L TMPT.

**Figure 3:**
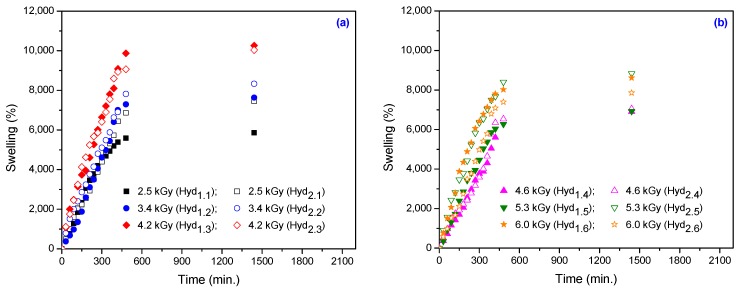
The effect of the amount of TMPT and PP on the swelling for hydrogels obtained at: (**a**) 2.5 kGy, 3.4 kGy and 4.2 kGy; (**b**) 4.6 kGy, 5.3 kGy and 6.0 kGy.

The water uptake in hydrogels at 24, 48 and 72 h, as a function of the absorbed dose and the amount of cross-linker and initiator, is shown in Figure 2. The hydrogels’ swelling isotherms, as a function of absorbed dose and amount of cross-linker, are shown in Figure 3.

As can be seen in Figure 2, the water uptake increases with the increase of the TMPT and PP amount at the same absorbed dose. Water uptake increases with the increase of the absorbed dose up to a maximum of 4.2 kGy and, after that, decreases with an increase in absorbed dose because the degree of cross-linking increases and the entrance of water is restricted. Also, the hydrogels with a greater amount of TMPT and PP (Hyd_2.1_–Hyd_2.6_) show a higher rate of swelling relative to that of hydrogels with a small amount (Hyd_1.1_–Hyd_1.6_), in the same time periods of swelling. This indicates that the hydrogels Hyd_2.1_–Hyd_2.6_ are characterized by good swelling properties relative to Hyd_1.1_–Hyd_1.6_. The maximum swelling was achieved at 48 h. Also, the differences between water uptake values at 48 and 72 h are very low, so that 48 h are sufficient to achieve a maximum degree of swelling.

In Figure 3 it can be seen again that the swelling increases with the increase in TMPT and PP amount at the same absorbed dose up to a certain point when it becomes constant. It can be noticed that, for the sample that contains a higher amount of TMPT (5.90 × 10^–3^ mol/L), even at the highest irradiation dose (6.0 kGy), the swelling is around 8500%, compared with the sample irradiated at the same dose, but with a lowest content of TMPT (2.95 × 10^–3^ mol/L) for which the swelling is almost 7400%. Once again, the swelling is strictly dependent on the absorbed dose and decreases when the degree of cross-linking increases.

#### 3.1.3. Equilibrium Water Content

The percentage of equilibrium water content (EWC%) is another parameter used for the assessment of hydrogels swelling and can be calculated using the following equation [34,40]:

(4) $$EWC=\frac{M_{S}-M_{0}}{M_{S}}\times 100$$

where *M_S_* is the mass of the swollen gel at equilibrium and *M*_0_ is the mass of the dried gel at time *t* = 0.

**Figure 4:**
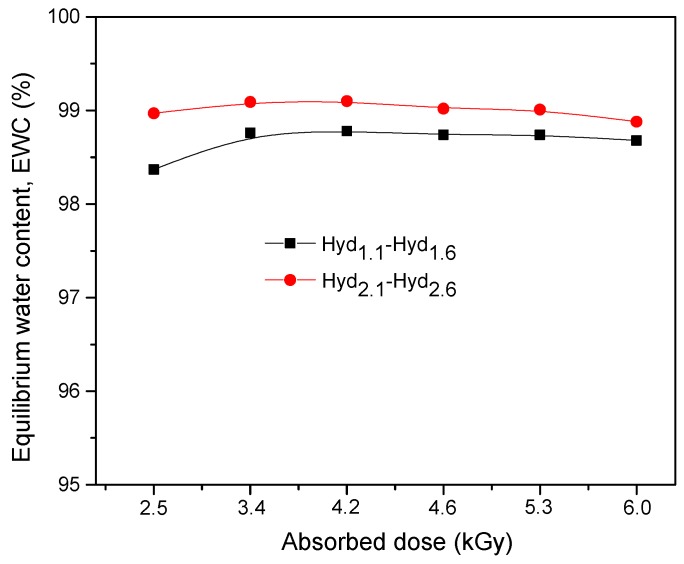
The effect of the absorbed dose and amount of TMPT on the equilibrium water content of hydrogels.

The results are presented in Figure 4, where all samples shows values of EWC% over 98%, even for high irradiation doses. At the same absorbed dose, the EWC% values of hydrogels that contain high amounts of TMPT are larger compared to those that contain small amounts. This occurs because TMPT is a hexafunctional cross-linker and its use in large amounts may form several bonds between chains [34].

It is known that water holding capacity and permeability are the most important features of a hydrogel. The polar hydrophilic groups are the first to be hydrated upon contact with water, which leads to the formation of primary bound water. As a result, the network swells and exposes the hydrophobic groups, which are also capable of interacting with the water molecules. This leads to the formation of hydrophobically-bound water, also called ‘secondary bound water’. Primary and secondary bound water are often combined and called ‘total bound water’. The network will absorb additional water due to the osmotic driving force of the network chains towards infinite dilution. This additional swelling is opposed by the covalent or physical cross-links, leading to an elastic network retraction force. Thus, the hydrogel will reach an equilibrium swelling level. The additional absorbed water is called ‘free water’ or ‘bulk water’ and is assumed to fill the space between the network chains and/or the center of larger pores, macropores, or voids. Depending on the nature and composition of the hydrogel, the next step is disintegration and/or dissolution if the network chains or cross-links are degradable.

#### 3.1.4. Swelling Kinetics

In order to examine the controlling mechanism of the swelling processes, several kinetic models are used to test the experimental data. A simple kinetic analysis is the following first-order equation [39]:

(5) $$\frac{dS}{dt}=k_{1,S}\left(S_{\max.}-S\right)$$

where *k*_1,*S*_ is the rate constant of first-order swelling and *S*_max._ is the degree of swelling at equilibrium.

After the integration by applying the initial condition (*S* = 0 at *t* = 0 and *S* = *S* at *t* = *t*), the equation becomes:

(6) $$\ln W=k_{1,S}t,\;\mathrm{where}\;W=\frac{S_{\max.}}{S_{\max.}-S}$$

A second-order equation based on swelling equilibrium degree is expressed by the following equation [39]:

(7) $$\frac{dS}{dt}=k_{2,S}{\left(S_{\max.}-S\right)}^{2}$$

where *k*_2*,S*_ is the rate constant of second-order swelling.

After integration and applying the initial condition (*S* = 0 at *t* = 0 and *S* = *S* at *t* = *t*), the equation becomes:

(8) $$\frac{t}{S}=A+Bt$$

where *A* is the reciprocal of the initial swelling rate and *B* is the inverse of the degree swelling at equilibrium:

(9) $$A=r_{0}=\frac{1}{k_{2,S}\times {S_{\max.}}^{2}}$$

(10) $$B=\frac{1}{S_{\max.}}$$

**Figure 5:**
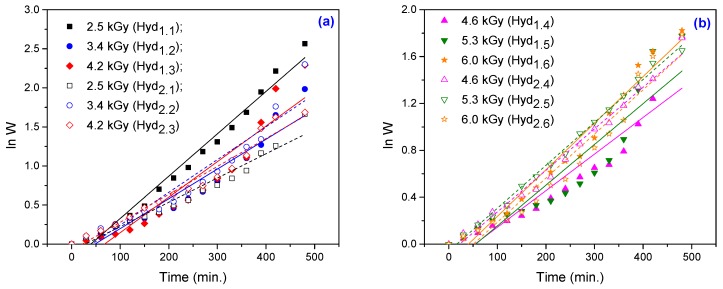
The first-order swelling kinetics for hydrogels obtained at: (**a**) 2.5 kGy, 3.4 kGy and 4.2 kGy; (**b**) 4.6 kGy, 5.3 kGy and 6.0 kGy.

**Figure 6:**
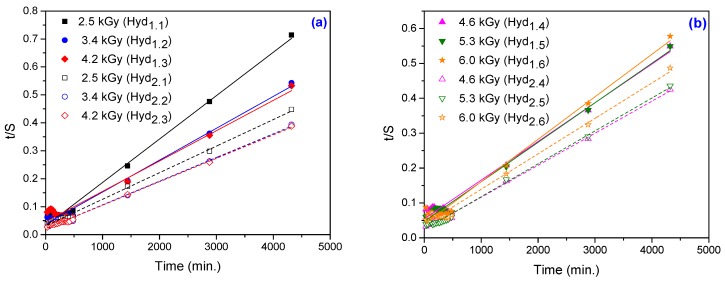
The second-order swelling kinetics for hydrogels obtained at: (**a**) 2.5 kGy, 3.4 kGy and 4.2 kGy; (**b**) 4.6 kGy, 5.3 kGy and 6.0 kGy.

By fitting the experimental data using the first-order equation (Figure 5) and the second-order equation (Figure 6), we can calculate the swelling kinetic parameters including the rate constant of first- and second-order swelling (*k*_1,*S*_ and *k*_2,*S*_), the theoretical degree swelling at equilibrium (*S*_max._) and the initial swelling rate (*r*_0_), from the slope and intercept of lines. The results are listed in Table 3 and Table 4.

In Table 3 we can see that the swelling rate constants do not decrease with an increase in the amount of TMPT. This suggests that the water diffusion at higher TMPT concentrations is not hindered due to steric obstacles caused by association, which leads to a denser gel structure, as was observed in the case of hydrogels based on polysaccharides [41].

As seen in Table 4, the initial swelling rate constant (*r*_0_) of the hydrogels has rapidly increased with the increase in absorbed dose for lower TMPT concentrations. At the same absorbed dose, *r*_0_ is smaller for samples that contain a higher amount of cross-linker. The swelling properties of hydrogels containing cross-linkers are changed because the molecules of cross-linkers are placed between the chains of monomers [42]. TMPT has a high degree of functionality, rapidly undergoes addition reactions and increases the cross-linking degree of the hydrogels. The maximum equilibrium swelling ratios theoretically calculated are in good agreement with the equilibrium swelling ratios obtained experimentally (Figure 3).

#### 3.1.5. Determination of Swelling Power

The analysis of water diffusion mechanisms in hydrogels clarifies polymer behavior and becomes important in the development of applications in biomedicine, pharmaceuticals, as well as environmental and agricultural engineering. The hydrogel swells when it is in contact with water because water diffuses, i.e., migrates into preexisting or dynamically formed spaces between the chains of hydrogels. The swelling of hydrogels involves a larger scale segmental motion, resulting, ultimately, in an increased separation between hydrogel chains. By exploiting the swelling experiment, the diffusion of water into the hydrogel can be determined. Also, by applying the following equations to 60% of swelling curves, the nature of the diffusion of water into hydrogels can be evaluated [42,43]:

(11) $$F_{swp}=\frac{M_{t}-M_{0}}{M_{0}}=kt^{n}$$

(12) $$\ln F_{swp}=n\ln t+\ln k,$$

where *M_t_* and *M*_0_ are the masses of the swollen sample at time *t* and of the dry sample, respectively; *k* is the swelling constant; and *n* is the swelling exponent, which is indicative of the transport mechanism.

The swelling–time curves of hydrogels in water are used to calculate the diffusion coefficients (*D*) by the short time approximation method. This method is valid only for the first 60% of the swelling [39]. The diffusion coefficients have been calculated using the following equation:

(13) $$F=4{\left[\frac{D}{\pi \times r^{2}}\right]}^{1/2}t^{1/2},$$

where *D* is in cm^2^·sec^−1^, *t* in sec and *r* is the radius of the cylindrical polymer sample (cm).

**Figure 7:**
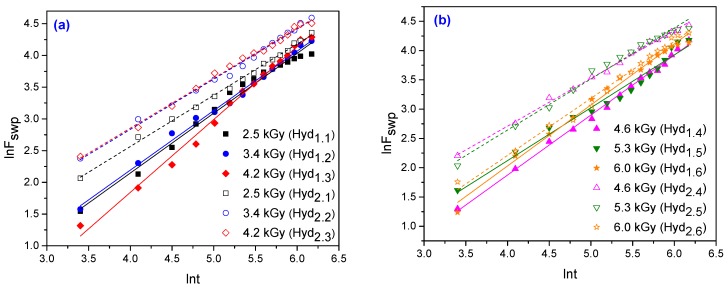
The plots of ln*F* versus ln*t* for hydrogels obtained at: (**a**) 2.5 kGy, 3.4 kGy and 4.2 kGy; (**b**) 4.6 kGy, 5.3 kGy and 6.0 kGy.

**Figure 8:**
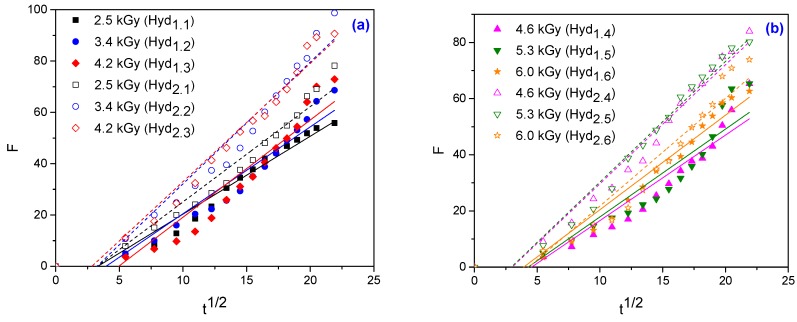
Plots of F versus *t*^1/2^ for hydrogels obtained at: (**a**) 2.5 kGy, 3.4 kGy and 4.2 kGy; (**b**) 4.6 kGy, 5.3 kGy and 6.0 kGy.

For the hydrogels obtained in this study, ln*F* versus ln*t* are plotted and shown in Figure 7 and the graphs of *F* versus *t*^1/2^ are shown in Figure 8. The swelling constant (*k*), swelling exponent (*n*) and diffusion coefficients (*D*) are calculated from the slopes and intercepts of the lines, respectively and are listed in Table 5.

According to the relative rates of diffusion (R_diff_) and relaxation (R_relax_), there are three classes of diffusion [44,45,46]: Case I: *n* = 0.45–0.5 indicates a Fickian diffusion mechanism, in which the rate of diffusion is much smaller than the rate of relaxation (R_diff_ << R_relax_) and the system is controlled by diffusion; Case II: *n* = 1.0, where the diffusion process is much faster than the relaxation process (R_diff_ >> R_relax_) and the system is controlled by relaxation; Case III: 0.5 < *n* < 1.0 indicates non-Fickian (anomalous) diffusion mechanism, which describes those cases where the diffusion and relaxation rates are comparable (R_diff_ ≈ R_relax_). Occasionally, when *n* > 1, the situation is regarded as Super Case II kinetics [44,47,48].

In Table 5 it can be noted that the values of the swelling coefficient (*n*) vary between 0.7 and 1.15. Based on the results, it can be said that the diffusion of water into the obtained hydrogels shows a non-Fickian character, with two exceptions: Hyd_1.3_ and Hyd_1.4_, for which the diffusion of water shows Super Case II kinetics. When the diffusion type involves anomalous behavior, the relaxation and diffusion times are of the same order of magnitude. For a non-Fickian diffusion mechanism, the cross-linking density is high, thus leading to a small amount of bulk water and a decreased diffusion rate [49,50,51]. It is known that the high water content of hydrogels is represented by the bulk water, which is similar with the bulk water coming from outside of the gel. In a polymeric network of hydrogels, there are at least three types of water structures, as follows: bulk water, primary and secondary bound water. When a hydrogel is immersed in water, the first type of water that will be present is the primary bound water, due to the hydration of hydrophilic groups of the polymer. The primary bound water is very difficult to remove from the gel. As a result, the network swells and the hydrophobic groups are exposed, leading to the formation of secondary bound water [51].

For two of the samples (Hyd_1.3_ and Hyd_1.4_), the swelling exponent (*n*) is higher than 1.0, which indicates that the transport mechanism is Super Case II or Case II (relaxation controlled). Penetration of water molecules is much greater than in the relaxation processes. The swelling exponent (*n*) slightly increased as a function of absorbed dose (up to 4.2 kGy) and decreased as the amount of TMPT increases. The diffusion coefficient (*D*) has the same tendency: it increases as a function of absorbed dose up to 4.2 kGy, followed by a slightly decrease, but increases as the amount of TMPT increases. This phenomenon has been attributed to the ease with which water molecules can diffuse into a hydrogel network [51].

#### 3.1.6. Network Studies

Directly related to the polymer cross-link density is *M_c_*, the average molar mass between cross-links, which is determined using the swelling equilibrium. According to the theory of Flory and Rehner for a perfect network, *M_c_* is calculated using the following equation [39]:

(14) $$M_{c}=-V_{1}d_{p}\frac{\nu_{S}^{1/3}-\nu_{S}/2}{\ln(1-\nu_{S})+\nu_{S}+\chi \nu_{S}^{2}}$$

where *V*_1_ is the molar volume of the solvent (in this case water: 18.1 cm^3^·mol^−1^); *d_P_* is the polymer density (1.106 g·cm^−3^); *υ_S_* is the volume fraction of the polymer in the swollen gel (cm^3^) and is equal to 1*/S*; and *χ* is the Flory–Huggins interaction parameter between the solvent and polymer.

The value of *χ* is calculated as follows [52,53,54]:

(15) $$\chi =0.431-0.311\times \nu_{S}-0.036\nu_{S}^{2}$$

The cross-link density *q* is defined as being the mole fraction of the cross-linked units:

(16) $$q=\frac{M_{0}}{M_{c}}$$

where *M*_0_ is the molecular weight of the repeating units from polymer and is calculated by the following equation [52,54]:

(17) $$M_{0}=\frac{\left(m_{AMD}\times M_{AMD})+(m_{AA}\times M_{AA})+(m_{TMPT}\times M_{TMPT}\right)}{m_{AMD}+m_{AA}+m_{TMPT}}$$

where *m_AMD_*, *m_AA_* and *m_TMPT_* are the masses of acrylamide, acrylic acid and cross-linker (TMPT), expressed in grams; and *M_AMD_*, *M_AA_* and *M_TMPT_* are the molar masses of acrylamide, acrylic acid and TMPT, expressed in g·mol^−1^.

Other important parameters used for the assessment of networks are gel pore or mesh size (*ξ*) and porosity (*P%*). The mesh size is related to the space available for the transport of a solute or solvent in a network. An increase in the mesh size and porosity results in an increase in the water content in the hydrogel. Using the calculated values of number average molecular mass between cross-links, *M_c_*, the mesh size, was determined by the following equation [55]:

(18) $$\xi =\nu_{S}^{-1/3}\times l\times \sqrt{\frac{2\times C_{n}\times M_{c}}{M_{0}}}$$

where *υ_S_* is the volume fraction of the polymer in the swollen gel, *l* is the length of the C–C bond along the polymer backbone (0.154 nm), *C_n_* is the Flory characteristic ratio of the polymer and *M_r_* is the molecular mass of the repeated unit. The characteristic ratio *C_n_* for poly(AMD-co-AA) hydrogels was considered as the weighted average of *C_n_* values for poly(AMD) and poly(AA) chains, according to their molar ratio in the hydrogel (*C_n_* was 8.8 for poly(AMD) and 6.7 for poly(AA)).

The porosity *P*(%) of the obtained hydrogels was determined using the following equation [39]:

(19) $$P(\%)=\frac{V_{d}}{1-V_{d}}\times 100,$$

where *V_d_* is the volume ratio of water at equilibrium.

The values of the number-average molar mass between cross-links (*M_c_*/g·mol^−1^), cross-link density (*q*), mesh size (*ξ*) and porosity (*P*) are shown in Table 6.

The average molecular weight between cross-links (*M_c_*), cross-linking density (*q*) and the network mesh size (*ξ*) determined from equilibrium swelling experiments are major parameters in defining the structure of a cross-linked hydrogel network.

*M_c_* features the number average molecular weight of polymer chains between two adjacent cross-link junctions. This parameter allows the degree of cross-linking of hydrogel samples to be measured and expressed as *q*. As is shown in Table 6, the number-average molar mass between cross-links of hydrogels has increased with the increase in the amount of cross-linker (TMPT). For both TMPT amounts, with the increasing of the irradiation dose, Mc increased up to a maximum at 4.2 kGy and then decreased. Due to the increase of the cross-linker concentration, the *M_c_* between the two main backbones increases. A large value of *M_c_* indicates long chains between the two backbones. The obtained results show that the *M_c_* values are affected by the absorbed dose. The increase in absorbed doses leads to a decrease in *M_c_* since the hydrogel becomes more and more dense. However, AMD includes several hydrophilic fragments and thus the hydrogels shows a high degree of swelling. The values of the cross-link density are inversed due to the value of number-average molar mass between cross-links. An increase in the amount of cross-linker increases the cross-linking density and decreases the swelling (Figure 2). This confirmed the role of TMPT as a cross-linking agent. The mesh size (*ξ*), sometimes referred as pore size, indicates the distance between two adjacent cross-links. It is critical in controlling the water or aqueous solutions of various salts and heavy metals’ diffusion rate as it reflects the amount of space available for a salt or heavy metal molecule to diffuse in or out of the swollen hydrogel network. The mesh size and porosity have decreased with the increase in the amount of cross-linker (TMPT) and the absorbed dose. More than that, from the results obtained it can be observed that the degree of cross-linking had a significant influence on the mesh size. Hydrogels having a higher degree of cross-linking have a relatively shorter distance between two cross-linking points and, as a result, the mesh sizes and porosity of these hydrogels are lower [55].

#### 3.1.7. Spectral Characterization

**Figure 9:**
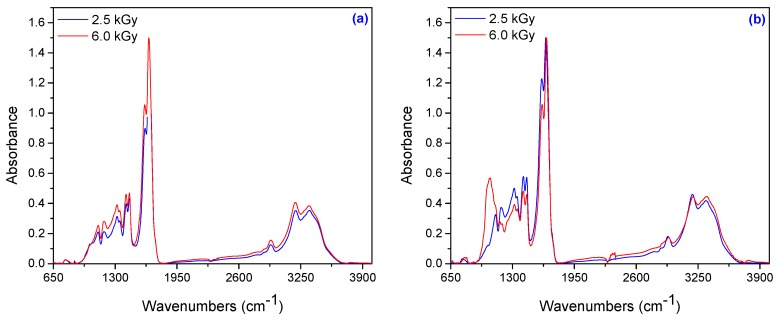
FTIR spectra in the range of 650–4000 cm^-1^ for samples obtained at 2.5 kGy and 6.0 kGy: (**a**) 3.70 × 10^−3^ mol/L PP and 2.95 × 10^−3^ mol/L TMPT; (**b**) 7.40 × 10^−3^ mol/L PP and 5.90 × 10^−3^ mol/L TMPT.

To understand the binding of AMD, AA and TMPT in hydrogels during irradiation, we evaluated the FTIR spectra of hydrogels obtained at 2.5 and 6.0 kGy (Figure 9). The broad bands in the range of 3345 to 3330 cm^−1^ are attributed to the symmetric and asymmetric –NH stretching vibrations of AMD and the broad bands in the region of 3200–3190 cm^−1^ are characteristic of the absorptions of –OH groups of AA. The methylene group vibrations are used to monitor the extent of polymerization. The absorbance in the range 2937–2933 cm^−1^ is assigned to the asymmetrical stretching vibrations of –CH_2_ from AMD or AA [56,57]. The bands in the range 1774–1785 cm^−1^ correspond to the carbonyl stretching vibration of C=O connected to the carboxyl group, but in the range of 1647–1657 cm^−1^ can be attributed to the C=O group connected to the amide group [56].

The characteristic band that appeared in the range of 1600–1620 cm^–1^ is due to the amide II bands. Amide II results from the N–H bending vibration and from the C–N stretching vibration. Symmetric stretching of COO^−^ is found at 1450–1410 cm^−1^ in the poly(acrylamide-*co*-acrylic acid) spectra [56]. On the other hand, the bands at 1443 cm^−1^ and 1353 cm^−1^ are attributed to CH_2_ bending and –CH bending vibrations, respectively. For the polyacrylamide, the –CN stretching appears at 1350–1345 cm^−1^ and –C–O stretching at 1125–1116 cm^−1^. The C–O–C asymmetric stretching and C–O stretching from AA are confirmed with the absorptions around 1190–1180 cm^−1^ and 1065–1025 cm^−1^, respectively [58].

#### 3.1.8. SEM Analysis

**Figure 10:**
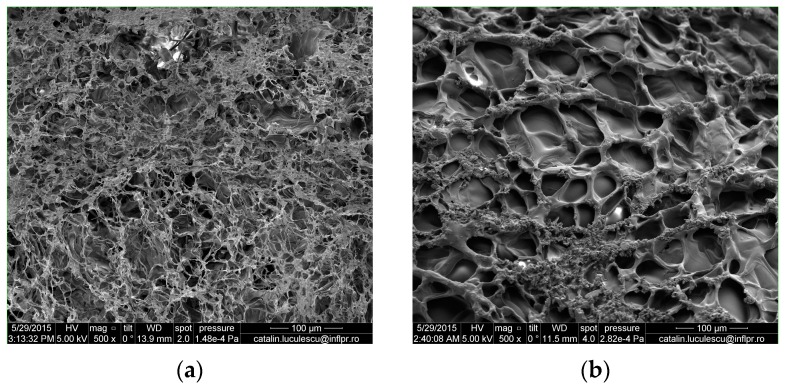
Scanning electron micrographs of hydrogels with 3.70 × 10^−3^ mol/L PP and 2.95 × 10^−3^ mol/L TMPT obtained by electron beam irradiation at (**a**) 2.5 kGy and (**b**) 6.0 kGy.

**Figure 11:**
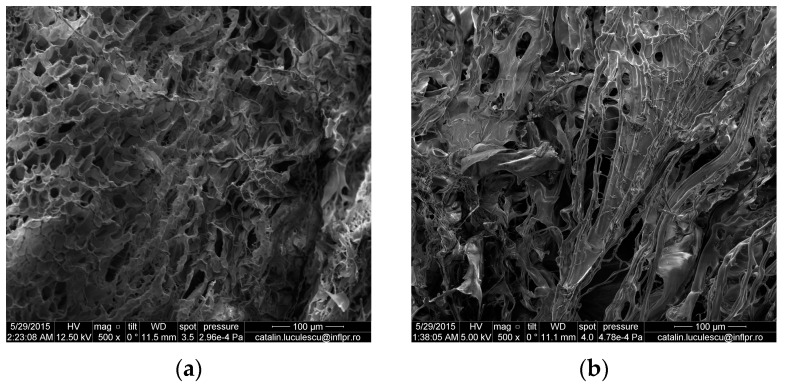
Scanning electron micrographs of hydrogels with 7.40 × 10^−3^ mol/L PP and 5.90 × 10^−3^ mol/L TMPT obtained by electron beam irradiation at (**a**) 2.5 kGy and (**b**) 6.0 kGy.

The technique of Scanning Electron Microscopy was used for observing the surface appearance and the structure of copolymers. The scanning electron micrographs of hydrogels obtained at 2.5 kGy and 6.0 kGy irradiation doses are shown in Figure 10 and Figure 11.

The cross-link density and composition affect the microstructure of hydrogels, which influences the swelling characteristics. In Table 6 it is observed that the cross-link density has increased with the increase in the amount of PP, TMPT and absorbed dose. On the other hand, the mesh size has decreased with the increase in the amount of PP, TMPT and absorbed dose. So, as can be seen in Figure 10 and Figure 11 (a and b), the hydrogels with a small amount of PP and TMPT (3.70 × 10^−3^ mol/L and 2.95 × 10^−3^ mol/L TMPT, respectively) have presented a more open and porous channel structure than the hydrogels with a high content of PP and TMPT (7.40 × 10^−3^ mol/L PP and 5.90 × 10^−3^ mol/L TMPT, respectively) for the same irradiation dose. These pores facilitate the transport of water [59,60,61] and the hydrogels Hyd_1.1_ and Hyd_1.6_ obtained at 2.5 kGy and 6 kGy, respectively, show the highest equilibrium water uptake (14,200% and 12,100%, respectively). With the increase in the amounts of PP and TMPT, the morphology of the hydrogels is changing (Figure 11). The SEM micrographs of the hydrogels obtained at 2.5 kGy and 6 kGy (Hyd_2.1_ and Hyd_2.6_) present an irregular surface with a semi-porous structure, with macro- and micropores within the hydrogels and show the lowest equilibrium water uptake (8200% and 7400%, respectively).

### 3.2. Metal Ion Uptake Studies

Uptake experiments of metal ions at different pH were carried out by placing for 72 h at room temperature (25 ± 0.1 °C) portions of 0.5 g of dried hydrogels in a series of flasks containing 100 mL aqueous solutions of Cu^2+^ and Cr^6+^ with an initial concentration of 500 mg·L^–1^. The pH was adjusted using 0.1 M NaOH and 0.1 M HCl. The effect of a heavy metal solution (Cu^2+^ and Cr^6+^) on the swellability of the investigated hydrogels (Hyd_1.1_–Hyd_1.6_ and Hyd_2.1_–Hyd_2.6_) was studied.

pH is an important parameter for hydrogels in the adsorption of metals ions. It affects the electronic status of the pendant functional groups, such as protonation–deprotonation of the basic groups, as well as dissociation–association of the acidic groups. In addition, it may also modify the oxidation form of the metal ions present in the medium [62].

**Figure 12:**
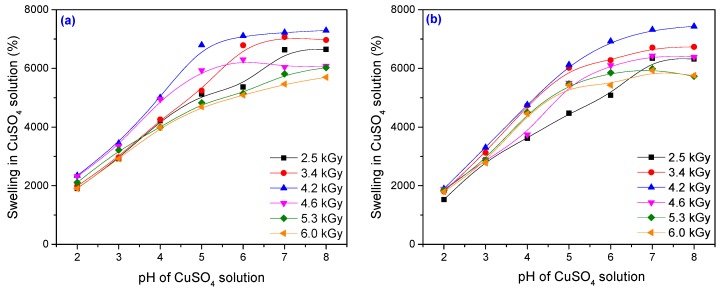
Swelling of the hydrogels in CuSO_4_ solution as a function of pH, PP and TMPT amount: (**a**) 3.70 × 10^−3^ mol/L PP and 2.95 × 10^−3^ mol/L TMPT; (**b**) 7.40 × 10^−3^ mol/L PP and 5.90 × 10^−3^ mol/L TMPT.

**Figure 13:**
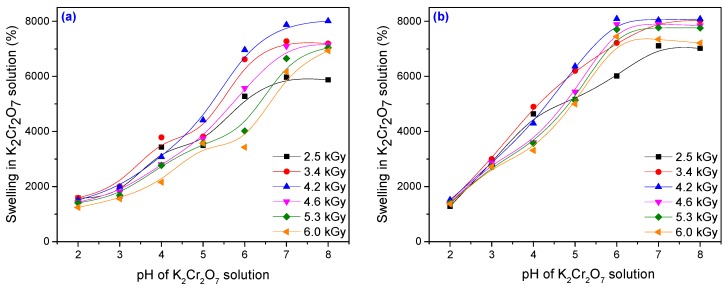
Swelling of the hydrogels in K_2_Cr_2_O_7_ solution as a function of pH, PP and TMPT amount: (**a**) 3.70 × 10^−3^ mol/L PP and 2.95 × 10^−3^ mol/L TMPT; (**b**) 7.40 × 10^−3^ mol/L PP and 5.90 × 10^−3^ mol/L TMPT.

The effect of pH on the swelling properties of the PAM–PAA hydrogel is shown in Figure 12 and Figure 13. The swelling values of hydrogels in a heavy metal solution of pH 7 is found to be significantly higher than those in a solution of pH 2. The swelling mostly occurred from pH 3 to 7. It is well known that the swelling of the hydrogel is induced by the electrostatic repulsion of the ionic charges of its network [63].

At pH > 7 the change in equilibrium swelling ratio with pH was superficial. A fully hydrated gel sample was almost 80 times heavier than a dried gel for pH > 6. At pH < 3 the degree of swelling was very low. When the hydration was carried out in a solution of pH < 3, the carboxylic acid groups on the copolymer backbone were converted to the protonated acid form [64]. At an acidic pH, most of the carboxylate anions are protonated, so the main anion–anion repulsive forces are eliminated and consequently swelling values are decreased. In addition, there are H-bonding interactions between the carboxylic groups of acrylic acid and the amide groups of acrylamide. These H-bonding interactions result in the formation of a compact or tight structure that does not permit much movement of polymeric chains within the hydrogel network, which leads to a minimum swelling of the hydrogel [63]. A low swelling ratio indicated that the water content for the acid form of the hydrogel was low. When the solution pH was above 6, the carboxylic groups on the copolymer backbone were converted to the salt (basic) form and the maximum degree of swelling was achieved. When the hydration occurred in a solution within the pH range 3–6, an almost linear relationship between the swelling ratio and pH was observed. Within this pH range, the acid and salt forms of the carboxylic groups on the copolymer backbone are both present [64]. As the pH of the swelling medium increased, ionization of the carboxylic acid groups of the gel occurred. That resulted in a more hydrophilic polymer network and contributed to higher water absorption as the pH increased [63]. Generally, it can be observed that the swellability of the hydrogel decreased in the heavy metal solution relative to double-distilled water. In the case of a heavy metal solution, the observed decrease in the swellability can be explained on the basis of complex formation. The interaction between metal ions and both carboxylate and amino (of amide) groups through coordination gives birth to a highly cross-linked network with small free volume inside the gel [65].

In Figure 12 and Figure 13 it can be seen that for both types of metal ion solutions, the swelling has increased with the increase in the absorbed dose, reaching a maximum and then decreasing. The best results, meaning the highest swelling, were obtained for a sample irradiated at 4.2 kGy. After 72 h, the filtrate of samples Hyd_1__.__3_ and Hyd_2__.__3_ obtained at 4.2 kGy was measured for metal ion concentration using an atomic absorption spectrometer (AAS). Every value reported was the mean of five measurements and the standard deviation was less than 5%. The amount of metal ion adsorbed was calculated using the equation [66]:

(20) $$Q=\frac{C_{0}-C_{72}}{m}\times V$$

where *Q* is the adsorbed amount of heavy metal ions (mg/g); *C*_0_ and *C*_72_ are the concentrations of metal ions in aqueous phase before and after treatment (mg/L); *V* is the volume of the aqueous phase (*L*); and *m* is the amount of dry hydrogel (g).

**Figure 14:**
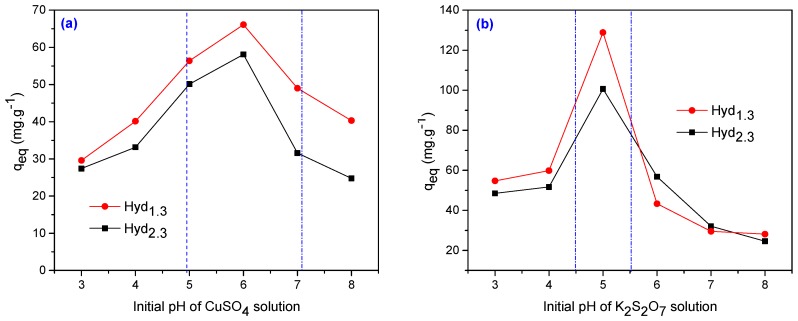
Effect of pH on (**a**) Cu^2+^ and (**b**) Cr^6+^ adsorption in Hyd_1.3_ and Hyd_2.3_ hydrogels (initial concentration: Cu^2+^ and Cr^6+^ 500 mg·L^−1^, dose: 0.5 g; volume 100 mL).

The adsorption of Cu^2+^ and Cr^6+^ in Hyd_1__.__3_ and Hyd_2__.__3_ hydrogels after 72 h atvarious pH values is represented in Figure 14.

Figure 14a clearly shows the effects of the initial pH (3.0–8.0) on the adsorption capacity of Cu^2+^ for both hydrogels (Hyd_1.3_ and Hyd_2.3_). From this figure, it is evident that the amounts of Cu^2+^ adsorption mainly depend on the pH values of the metal solution. The uptake of Cu^2+^ was found at a gradual increase from pH 3.0–4.0, beyond which the values sharply increase from 4.0 to 5.0 and the highest adsorption pH value is at 6.0. This may be because of the protonation of –NH_2_ from the acrylamide groups and the corresponding –OH in the acrylic acid under acidic conditions, generating repulsive charges between these functional groups and Cu^+2^ ions. The Cu^+2^ absorption at pH 6.0 was 66 mg/g for the Hyd_1.3_ (3.70 × 10^−3^ mol/L PP and 2.95 × 10^−3^ mol/L TMPT) and 58 mg/g for the Hyd_2.3_ (7.40 × 10^−3^ mol/L PP and 5.90 × 10^−3^ mol/L TMPT). The adsorption of Cr^6+^ on selected hydrogels at various pH values (3.0–8.0) was investigated and represented in Figure 8b. The adsorption increases with an increase in the pH value of the medium and a maximum recovery of Cr^6+^ was obtained in the acidic range, at a pH value at 5.0. Cr^6+^ exists in different forms in aqueous solution and the stability of these forms is dependent on the pH of the solution. In the pH range of 1 to 6, there are different forms of chromium ions such as Cr_2_O_7_^2–^, HCrO_4_^2–^, Cr_3_O_10_^2–^ and Cr_4_O_13_^2–^, while HCrO_4_^2–^ predominates in the solution [66,67]. Thus Cr^6+^ ions exist predominantly as HCrO_4_^2–^ in an acidic medium and the amino groups (–NH_2_) of acrylamide would be in protonated cationic form (–NH^3+^) to a higher extent in an acidic solution, which causes an electrostatic interaction between the hydrogel and HCrO_4_^2–^, resulting in high chromium removal [66,68]. At higher pH values, the decrease in the adsorption capacity can be associated with the appearance of Cr(OH)_3_ and anionic Cr(OH)^4–^ species due to the presence of OH^–^ ions and the uptake decreases. These species may have electrostatic repulsion with the deprotonated amine groups [69,70]. So, a pH of 5 was considered optimum for the removal of Cr^6+^ and the results were: 128 mg/g for Hyd_1.3_ (3.70 × 10^−3^ mol/L PP and 2.95 × 10^−3^ mol/L TMPT) and 102 mg/g for the Hyd_2.3_ (7.40 × 10^−3^ mol/L PP and 5.90 × 10^−3^ mol/L TMPT).

## 4. Conclusions

Poly(acrylamide co-acrylic acid) hydrogels were prepared by free-radical copolymerization of acrylamide (AMD) and acrylic acid (AA) in aqueous solutions, using potassium persulfate (PP) as initiator and trimethylolpropane trimethacrylate (TMPT) as cross-linker, by electron beam irradiation in the dose range of 2.5 kGy to 6 kGy. The influence of the absorbed dose and amount of cross-linker on the swelling properties, diffusion coefficient and network parameters of hydrogels was investigated. The gel content increases with the absorbed dose, cross-linking agent (TMPT) and initiator (PP) amount increase. Thus, about 90% gel content was attained at doses over 5.3 kGy. Water uptake increases with the increase of the absorbed dose, up to 4.2 kGy. The swelling increases with the increase of TMPT and PP amounts until the point where it becomes constant at the same absorbed dose. The percentage of equilibrium water content (EWC%) is over 98% and even higher for hydrogels containing large amounts of TMPT at the same irradiation dose. The best results, meaning the highest swelling, were obtained for a sample irradiated with 4.2 kGy. The swelling mostly occurred for pH values between 3 and 7. Over a pH of 7 the change in equilibrium swelling ratio with pH was slight. It was found that the use of a relatively small amount of TMTP for hydrogel preparation led to an increase in swelling up to 8500%. The water diffusion was non-Fickian and the number average molar mass between cross-links has increased with the absorbed dose and cross-linker content. The variation of the swelling kinetic parameters including the rate constant of first- and second-order swelling (*k*_1,*S*_ and *k*_2,*S*_), the theoretical degree swelling at equilibrium (*S_max._*) and the initial swelling rate (*r*_0_) with the amount of TMPT, PP and absorbed dose were evaluated. The variation of swelling constant (*k*), the swelling exponent (*n*) and the diffusion coefficients (*D*) with the amount of TMPT and absorbed dose are presented. The swelling exponent (*n*) slightly increased as a function of absorbed dose (up to 4.2 kGy) and decreased as the amount of TMPT increased. The diffusion coefficient (*D*) had the same tendency but increased with the amount of TMPT. The hydrogel network study was done by watching the variation of the number average molar mass between cross-links (M_c_/g mol× 10^−1^), cross-link density (q), mesh size (*ξ*) and porosity (*P*) with the amount of TMPT, PP and absorbed dose. The FTIR spectra of samples irradiated at 2.5 and 6 kGy validate the binding of AMD, AA and TMPT in the structure of the obtained hydrogels. Same sample micrographs present an irregular surface with semi-porous structure and with macro- and micro-pores correlated with the lowest equilibrium water uptake. The hydrogels’ efficiency in Cu^2+^ and Cr^6+^ removal from aqueous solutions was investigated at room temperature. During the adsorption of metal ions, residual metal ion concentration was measured in solution by atomic absorption spectrophotometry. The uptake of Cu^2+^ gradually increased from pH 3.0 to 4.0 and at pH 6.0 obtained the highest adsorption. pH 5 was considered optimum for removal of Cr^6+^.

## Figures and Tables

**Table 1:** The materials used for hydrogel preparation.

Materials	Chemical Characteristics	Chemical Structure
Acrylamide, AMD	molecular weight: 72.06 g/mol; density 1.051 g/cm^3^; solubility in water: 2.04 kg·L^−1^ at 25 °C	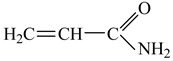
Acrylic acid, AA	molecular weight: 71.08 g/mol; density 1.13 g/cm^3^; soluble in water;	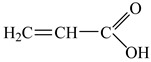
Potassium persulfate, PP (used as initiator)	molecular weight: 270.322 g/mol; density 2.477 g/cm^3^; solubility in water: 1.75 g/100 mL at 0 °C;	K_2_S_2_O_8_
Trimethylolpropane trimethacrylate, TMPT (used as cross-linker)	molecular weight: 338.4 g/mol; boiling point: >200 °C; density 1.06 g/cm^3^; 75 ± 3% active ingredient;	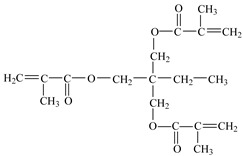

**Table 2:** Hydrogel synthesis details.

Samples Codes	Amount of Chemicals (mol/L)				Irradiation Dose (kGy)
	AMD	AA	PP	TMPT	
Hyd_1.1_	5	0.5	3.70 × 10^−3^	2.95 × 10^−3^	2.5
Hyd_1.2_					3.4
Hyd_1.3_					4.2
Hyd_1.4_					4.6
Hyd_1.5_					5.3
Hyd_1.6_					6.0
Hyd_2.1_	5	0.5	7.40 × 10^−3^	5.90 × 10^−3^	2.5
Hyd_2.2_					3.4
Hyd_2.3_					4.2
Hyd_2.4_					4.6
Hyd_2.5_					5.3
Hyd_2.6_					6.0

**Table 3:** The variation of first-order, *k*_1,*S*_/min^−1^ and second-order *k*_2,*S*_/g_gel_·(g_water_·min)^−1^ swelling rate constants with the amount of TMPT, PP and absorbed dose.

PP (mol/L)	TMPT (mol/L)	Absorbed Dose (kGy)					
		2.5	3.4	4.2	4.6	5.3	6.0
		***k*_1,*S*_ × 10^3^**					
**3.70 × 10^−3^**	**2.95 × 10^−3^**	5.42	3.81	4.55	3.12	3.48	3.98
**7.40 × 10^−3^**	**5.90 × 10^−3^**	3.11	4.19	3.58	3.55	3.67	3.78
		***k*_2,*S*_ × 10^7^**					
**3.70 × 10^−3^**	**2.95 × 10^−3^**	7.69	3.39	2.54	2.25	2.72	3.81
**7.40 × 10^−3^**	**5.90 × 10^−3^**	2.88	3.31	3.26	3.45	3.76	2.73

**Table 4:** The variation of initial swelling rate, *r*_0_/g_water_·(g_gel_·min)^−1^ and equilibrium swelling degrees (theoretical) *S*_max._/g_water_·(g_gel_)^−1^ with the amount of TMPT and absorbed dose.

PP (mol/L)	TMPT (mol/L)	Absorbed Dose (kGy)					
		2.5	3.4	4.2	4.6	5.3	6.0
		***r*_0_ × 10^2^**					
**3.70 × 10^−3^**	**2.95 × 10^−3^**	3.13	3.83	4.62	5.46	4.71	3.89
**7.40 × 10^−3^**	**5.90 × 10^−3^**	3.14	2.17	2.16	2.45	2.37	3.78
		***S*_max._**					
**3.70 × 10^−3^**	**2.95 × 10^−3^**	6440	8768	9215	9015	8820	8207
**7.40 × 10^−3^**	**5.90 × 10^−3^**	10,519	11,801	11,929	10,888	10,603	9843

**Table 5:** The variation of *n*, *k* and diffusional coefficient (*D* × 10^−3^/cm^2^·sec^−1^) with the amount of TMPT and absorbed dose.

PP (mol/L)	TMPT (mol/L)	Absorbed Dose (kGy)					
		2.5	3.4	4.2	4.6	5.3	6.0
		***n***					
**3.70 × 10^−3^**	**2.95 × 10^−3^**	0.94	0.93	1.15	1.02	0.90	0.98
**7.40 × 10^−3^**	**5.90 × 10^−3^**	0.81	0.79	0.79	0.82	0.87	0.97
		***k***					
**3.70 × 10^−3^**	**2.95 × 10^−3^**	0.20	0.21	0.06	0.11	0.23	0.12
**7.40 × 10^−3^**	**5.90 × 10^−3^**	0.51	0.72	0.75	0.57	0.43	0.18
		***D* × 10^3^**					
**3.70 × 10^−3^**	**2.95 × 10^−3^**	0.31	0.49	0.77	0.59	0.73	0.96
**7.40 × 10^−3^**	**5.90 × 10^−3^**	1.46	2.62	2.69	2.46	2.72	2.31

**Table 6:** The variation of the number-average molar mass between cross-links (M_c_/g·mol**^−^**^1^), cross-link density (*q*), mesh size (*ξ*) and porosity (*P*) with the amount of TMPT, PP and absorbed dose.

PP (mol/L)	TMPT (mol/L)	Absorbed Dose (kGy)					
		2.5	3.4	4.2	4.6	5.3	6.0
		***M_c_* × 10^−3^**					
**3.70 × 10^−3^**	**2.95 × 10^−3^**	179	184	190	176	179	164
**7.40 × 10^−3^**	**5.90 × 10^−3^**	625	668	684	582	559	458
		***q* × 10^4^**					
**3.70 × 10^−3^**	**2.95 × 10^−3^**	4.013	3.916	3.783	4.089	4.001	4.391
**7.40 × 10^−3^**	**5.90 × 10^−3^**	1.160	1.086	1.061	1.246	1.297	1.583
		***ξ*/nm**					
**3.70 × 10^−3^**	**2.95 × 10^−3^**	107	138	142	135	137	128
**7.40 × 10^−3^**	**5.90 × 10^−3^**	258	293	298	266	258	226
		***P*(%)**					
**3.70 × 10^−3^**	**2.95 × 10^−3^**	98.73	98.74	98.77	98.71	98.73	98.66
**7.40 × 10^−3^**	**5.90 × 10^−3^**	99.05	99.09	99.10	99.01	98.99	98.87
